# Supplementary figures and images for: A dynamic interplay between chitin synthase and the proteins Expansion/Rebuf reveals that chitin polymerisation and translocation are uncoupled in Drosophila
Source: PLoS Biol. 2023 Jan 23;21(1):e3001978. doi: 10.1371/journal.pbio.3001978 (PMC9894549; doi:10.1371/journal.pbio.3001978)

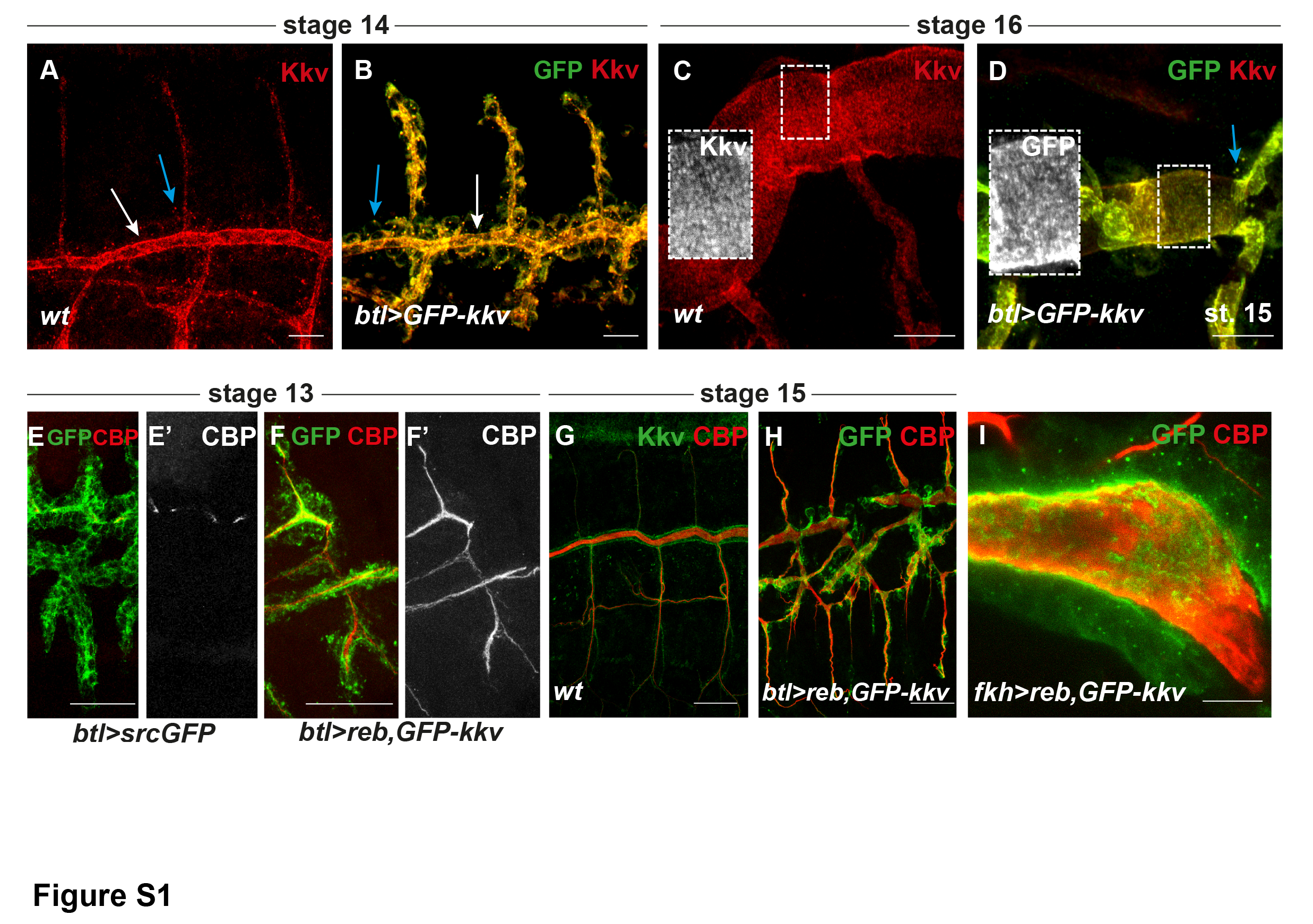

Supplement: S1 Fig — All images correspond to projections of confocal sections. (A-D) At early stages, overexpressed GFP-Kkv accumulates apically (white arrow in B) and in intracellular punctae (blue arrow in B), as endogenous Kkv (white and blue arrows in A), but also in the whole cell. At later stages, GFP-Kkv shows a pattern in stripes that corresponds to the taenidial folds (inset in D), in a comparable pattern to the endogenous Kkv (inset in C). Endogenous Kkv at late stages localises mainly apical and almost no intracellular punctae are detected (C). GFP-Kkv also localises mainly apical, but in addition, Kkv intracellular punctae are also detected (blue arrow in D). (E-H) In trachea, the simultaneous overexpression of reb and GFP-kkv anticipates chitin deposition (compare E and F). At later stages, this results in different morphogenetic defects like short and straight tubes and defects in branch fusion (compare H and G). (I) In salivary glands, the coexpression of reb and GFP-kkv promotes accumulation of chitin in the lumen. Scale bars: 10 μm. (TIF) [file pbio.3001978.s001.tif]

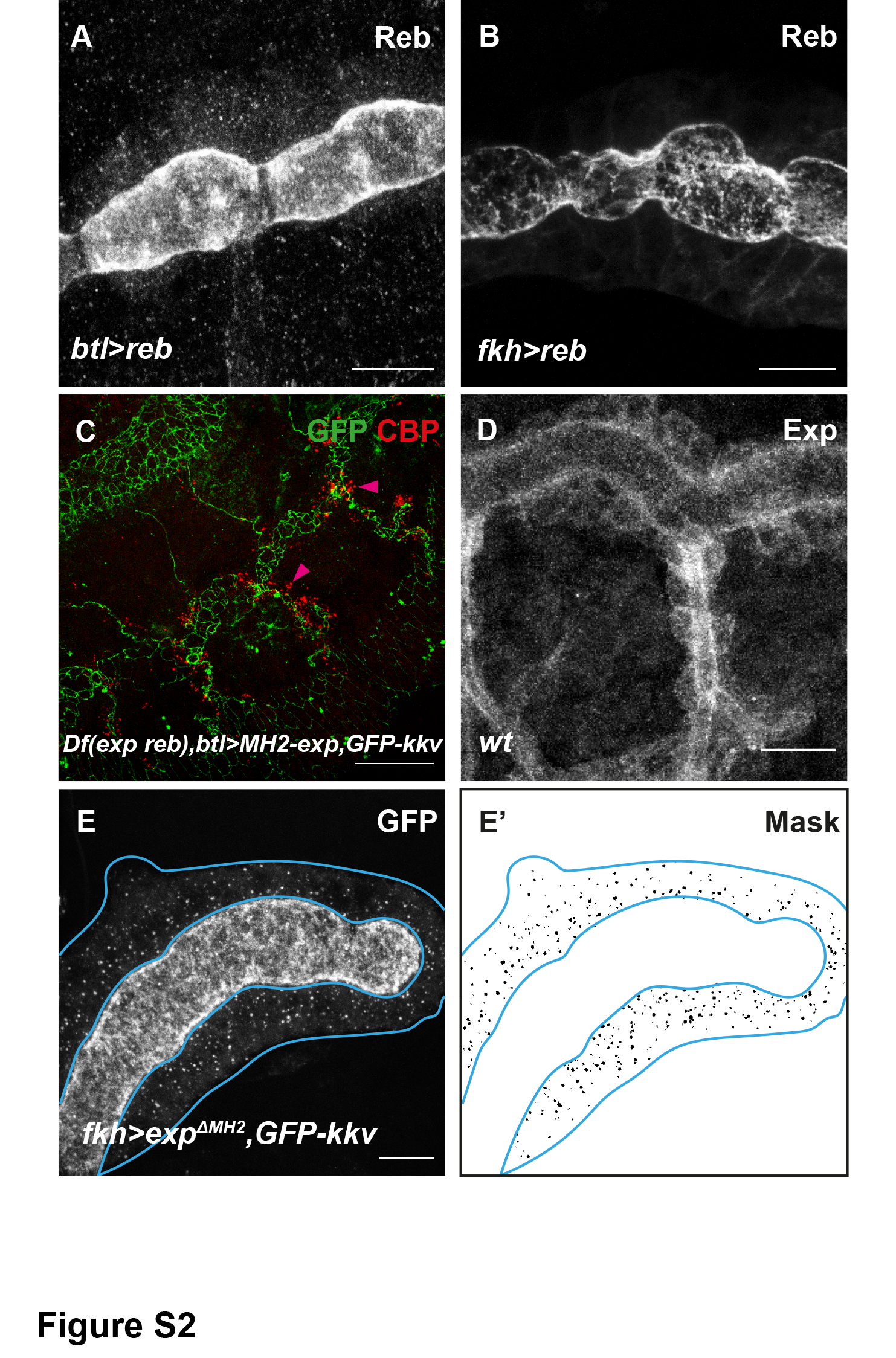

Supplement: S2 Fig — All images are projections of confocal sections. (A, B) Overexpressed full-length Reb localises mainly apically in trachea (A) and in salivary glands (B). (C) The simultaneous overexpression of MH2-exp and GFP-kkv does not rescue the absence of extracellular chitin deposition, and it produces intracellular chitin vesicles (pink arrowheads). (D) Endogenous Exp localises mainly apically in trachea, although a bit of the protein can be detected intracellularly. (E) Example of a salivary gland used to quantify the number of GFP-Kkv vesicles in Fig 5I. (E’) A mask to count the vesicles is generated by substracting background and the apical membrane region. Scale bars: 10 μm. (TIF) [file pbio.3001978.s002.tif]

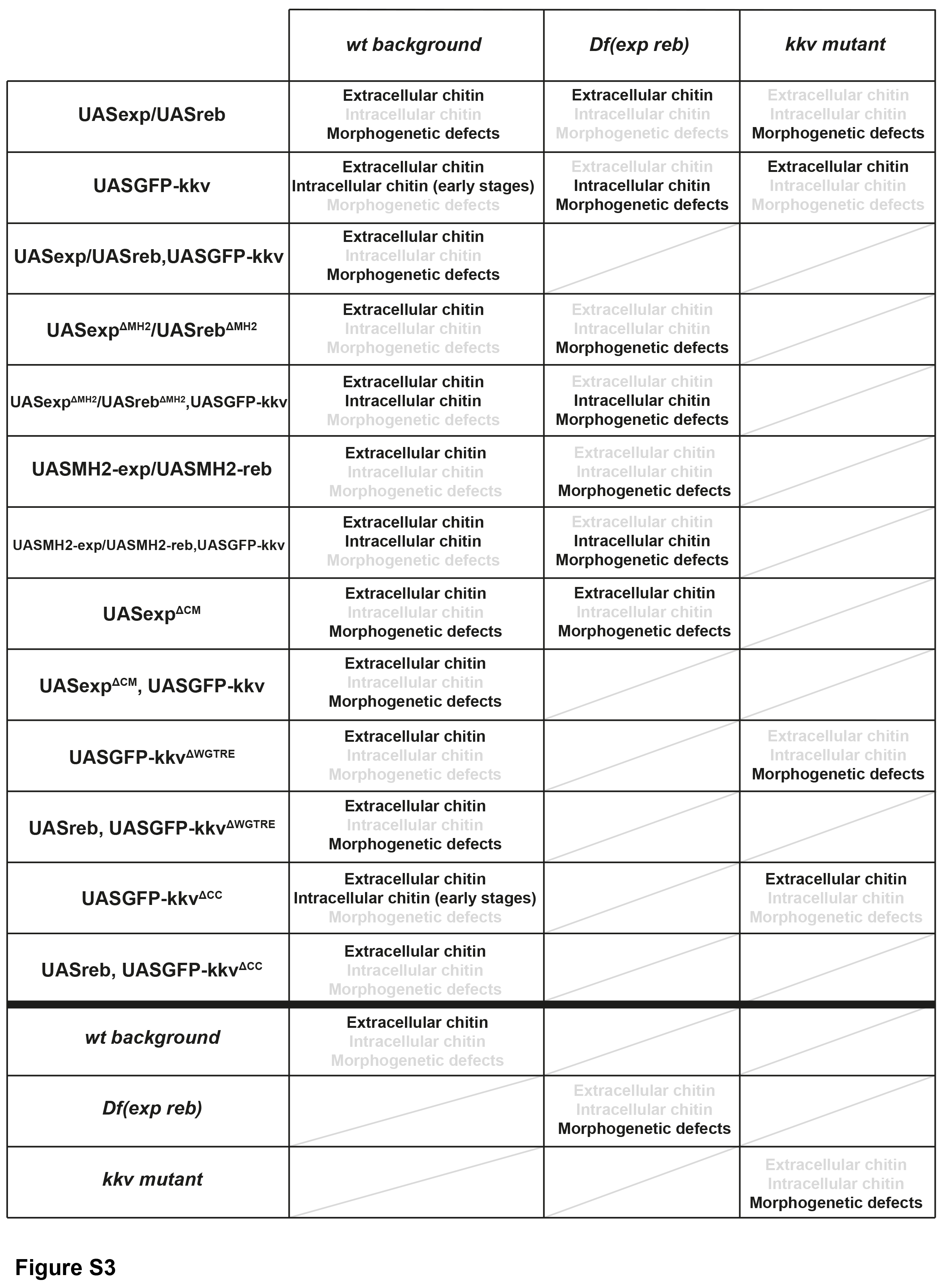

Supplement: S3 Fig — Summary of phenotypes of the different UAS constructs in wild-type (wt), exp reb mutant, and kkv mutant embryos in different overexpression conditions. The phenotypes observed are indicated in black, and light grey indicates absence of the phenotype (TIF) [file pbio.3001978.s003.tif]

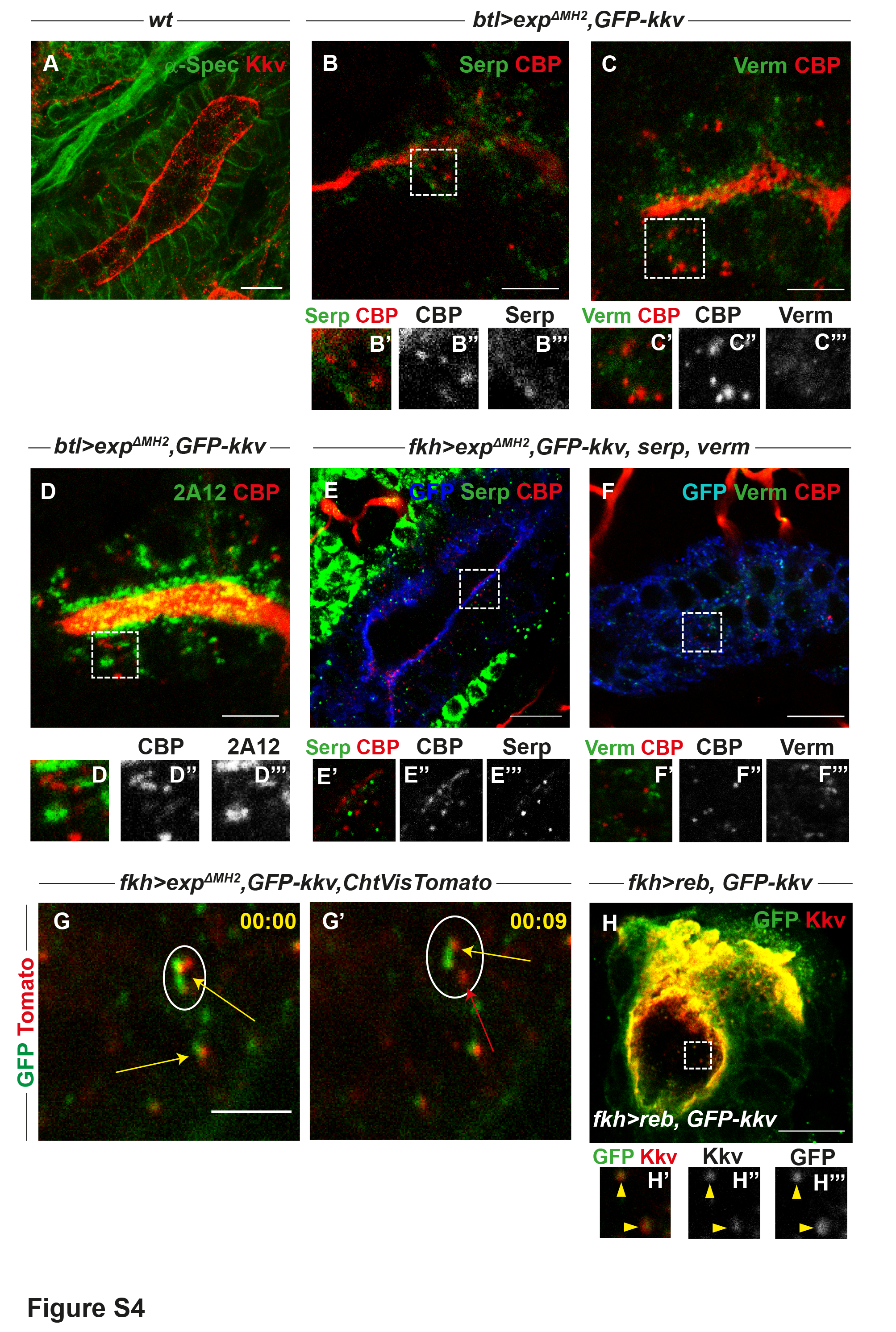

Supplement: S4 Fig — (A) Projection of confocal sections of salivary glands shows that Kkv is normally expressed in salivary glands and accumulates apically. (B-D) Single confocal sections of trachea at early stages. Single chitin punctae do not colocalise with deacetylases or Gasp. (E, F) Single confocal sections of salivary glands expressing serp and verm. Single chitin punctae do not colocalise with deacetylases. (G) Frames from live imaging movie 2 show that common GFP-Kkv and chitin punctae (yellow arrow) can separate from each other; many GFP-Kkv (green arrow) and chitin puncta (red arrow) do not colocalise. (H) The luminal GFP-Kkv punctae are labelled by GFP and Kkv. Scale bars A, E, F: 5 μm; C-D, G: 10 μm. (TIF) [file pbio.3001978.s004.tif]

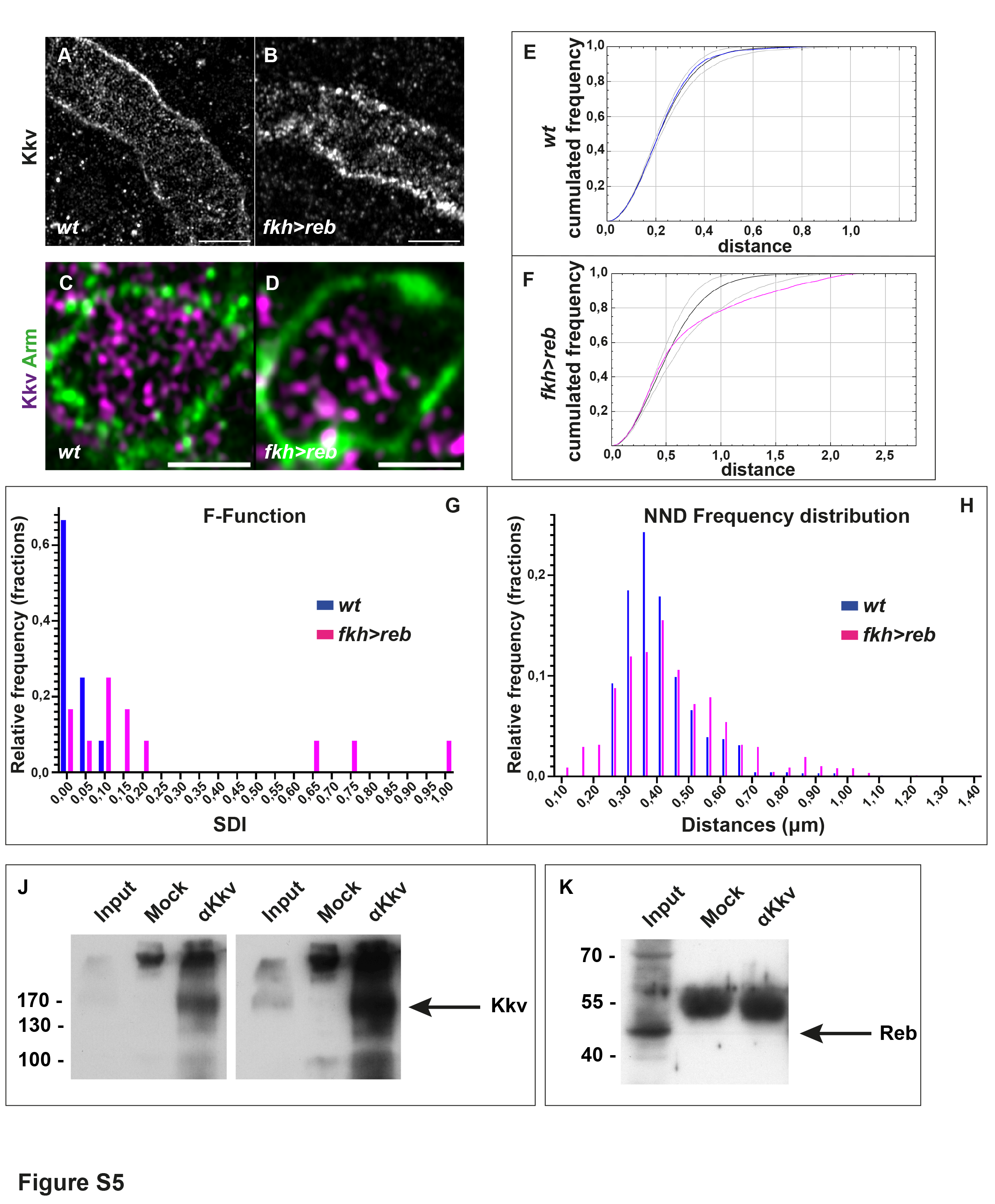

Supplement: S5 Fig — (A, B) Kkv distribution on the apical surface of a control (A) and an embryo expressing reb in salivary glands (fkhGal4>UAS-reb, B) and zoomed images for Kkv punctae (magenta) on the apical cell area marked by arm (green) of a control (C) and a fkhGal4>UAS-reb (D) embryo. The corresponding observed F function for the control (E) and the fkhGal4>UAS-reb (F) are displayed within and below the reference simulated random distributions (black) and the 95% confidence interval (light gray), respectively, indicating a random spatial pattern for the control and a tendency towards the formation of aggregates for the reb ectopic expression. (G) SDI histogram for the F-Function of the control (blue) and the fkhGal4>UAS-reb (magenta) samples. A significant difference between the frequency distributions for each group of individuals has been observed. (Kolmogorov–Smirnov D = 0.6667, p < 0.01) (H) Frequency distribution histograms for the Nearest Neighbour Distances (NNDs) between Kkv punctae in control (blue) and fkhGal4>UAS-reb samples. The distribution of values between the two groups is found significantly different (Kolmogorov–Smirnov D = 0.1463, p < 0.005). All images are projections of confocal sections, of super-resolution microscopy. The underlying data for quantifications can be found in the S1 Data. (J, K) Western blot using αKkv (J, two different exposure times are shown) or αReb (K) of embryo extracts that were subjected to immunoprecipitation with αKkv or an unrelated antibody (mock). Input correspond to 7.5% of the immunoprecipitated material. The position of MW markers (in kDa) is indicated. Scale bars A, B: 5 μm; C, D: 2 μm. (TIF) [file pbio.3001978.s005.tif]

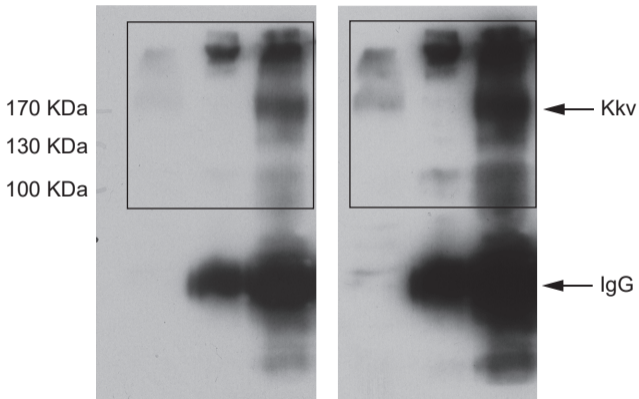

Original uncropped western blots for S5J Fig

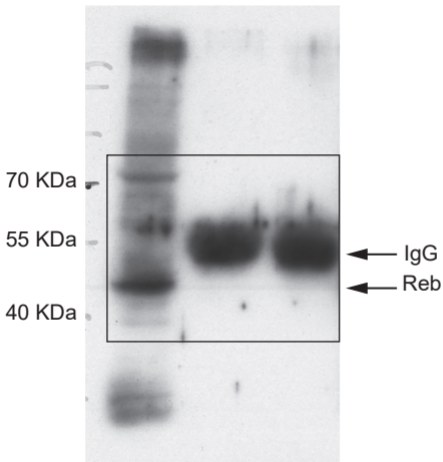

Original uncropped western blot for S5K Fig

Supplement: S1 Raw Images — The excerpted portion of the immunoblot shown in the figures is highlighted by a black box. (PDF) [file pbio.3001978.s009.pdf]
