# Supplementary material for: A dynamic interplay between chitin synthase and the proteins Expansion/Rebuf reveals that chitin polymerisation and translocation are uncoupled in Drosophila
Source: PLoS Biol. 2023 Jan 23;21(1):e3001978. doi: 10.1371/journal.pbio.3001978 (PMC9894549; doi:10.1371/journal.pbio.3001978)
Supplement: Attachment — Submitted filename: 128994_1_rebuttal_2495319_rk1q50.docx [file pbio.3001978.s010.docx]

**Manuscript number:** RC-2022-01598

**Corresponding author(s):** Marta Llimargas

*[Please use this template only if the submitted manuscript should be considered by the affiliate journal as a full revision in response to the points raised by the reviewers.*

*If you wish to submit a preliminary revision with a revision plan, please use our "*[*Revision Plan*](https://docs.google.com/document/u/0/d/1HHp62Tfeb44nd3Gu_3ChB0b8gmMdEog7GjZcZFwHSiQ/edit)*" template.* ***It is important to use the appropriate template to clearly inform the editors of your intentions.****]*

## 1. General Statements [optional]

In this work, we are advancing in our current understanding of the cellular and molecular mechanisms regulating chitin deposition. Chitin is a highly abundant polysaccharide in nature and a principal component of apical extracellular matrices in insects. Besides its physiological relevance, it is an excellent biomaterial with multiple applications nowadays, as well as a primary target for anti-fungal agents and pesticides. Understanding the mechanisms of chitin deposition is therefore a key goal both in biology and biotechnology.

Our manuscript has undergone extensive revision by three expert reviewers who have recognised its relevance. The reviewers state that this is a "*new and fundamental work of high significance*", which advances the current knowledge about chitin production. In addition, they consider this work to be of "*broad interest to the cell biology and developmental biology communities, and to the translational community working to develop chitin as a commercial biopolymer*".

We have addressed all the concerns raised by the reviewers, either by performing new experiments or by clarifying the text. We consider that the new additions and clarifications clearly improve our manuscript and give it the potential to represent a step-forward in the field.

## 2. Point-by-point description of the revisions

**Reviewer #1**

Major comments

**1.** Fig. 4 Does ectopic overexpression of Kkv-GFP have the same expression pattern as the endogenous Kkv? The overexpression line may lead to ectopic expression. the colocalization of endogenous Kkv and intracellular vesicles would be more accurate.

We agree with the reviewer that using overexpression lines may have some inconveniences. Actually, we already discussed this point in the first section of Discussion. However, we would like to argue that it also has some advantages and can provide an experimental setup to test the function of Exp/Reb and Kkv.

As a first point we should mention our observation that endogenous levels of Kkv are not sufficient to give rise to detectable intracellular chitin punctae. According to this finding, we would like to propose that Kkv endogenous levels are low or limiting and while these low amount of Kkv may be able to produce chitin, it would be under the level of detection with our staining approach. Taking into account that endogenous Kkv is not able to produce detectable intracellular chitin in a consistent way, we cannot perform the experiment that the reviewer is suggesting (analyse colocalisation of endogenous Kkv and intracellular chitin vesicles).

The overexpression of GFP-Kkv is the only condition where we can consistently detect chitin intracellularly, and we believe that our results validate the use of GFP-Kkv overexpression to analyse chitin synthesis and deposition as it works like endogenous Kkv. First, we find that GFP-Kkv expression is able to fully rescue the lack of chitin produced by the absence of kkv activity (Moussian et al, 2015). Second, we find that GFP-Kkv expression is not able to promote chitin deposition in the absence of Exp/Reb activity and does not produce dominant or artifactual effects. Finally, as requested by the reviewer, we find a comparable pattern of endogenous Kkv and overexpressed GFP-Kkv. Actually, GFP-Kkv overexpression produces, as expected, increased levels of the protein in the apical domain, in vesicles, and in the whole cell, particularly at early stages (A,B'). At later stages, overexpressed GFP-Kkv mainly localises to the apical membrane (D'), in a comparable pattern to the endogenous one (C). At late st 16, overexpressed GFP-Kkv localises in the stripes that correspond to the taenidial folds (F,F'), as endogenous Kkv does (E,E'), indicating that it localises in the wild type pattern. This data is now introduced in the first chapter of results and shown in Fig S1A-D.

Figure: Panels show projections of lateral views of embryos at the stages indicated. At stage 14, GFP-Kkv overexpression produce high levels in the apical domain, as endogenous Kkv, but also in the whole cell. At later stages, overexpressed GFP-Kkv localises mainly apically. Note accumulation in the taenidial folds, which indicates that GFP-Kkv accumulates in the same pattern as endogenous Kkv, and that overexpression of Kkv does not change the normal pattern of accumulation. Scale bar 10 μm

**2.** Are Kkv and Exp/reb expressed at the same time endogenously? If kkv is expressed earlier than Exp, can intracellular chitin be detected in wild-type embryos at early stages? Fig. 1b shows overexpression of Kkv at S13 has intracellular chitin (exp is not expressed at this stage).

As we have explained in the previous point, we cannot detect chitin intracellularly produced by endogenous Kkv. As suggested by the reviewer, we tried to visualise intracellular chitin in conditions of absence of exp/reb and presence of endogenous Kkv, for instance in exp reb mutants or in wild type embryos at st 12/13. However, in our experimental setting we could not clearly detect it. As discussed previously, we assume that this is due to low levels of endogenous Kkv. This is now discussed in the first chapter of results

**3.** Fig. 1B no intracellular chitin is detected. Fig. 1H intracellular chitin is detected. Does Overexpression of exp-MH2 interfere with the endogenous Exp function?

We thank the reviewer for this observation. We find some intracellular chitin punctae in the trachea at stages in which exp and reb are already expressed when we co-express kkv and exp^ΔMH2^/reb^ΔMH2^. This indicates that endogenous Exp/Reb are not sufficient to translocate the chitin produced by Kkv in these conditions (while they are when only Kkv is overexpressed). This result, as the reviewer suggests, could indicate that the mutant proteins interfere with the normal activity of Exp and Reb. Because we propose that Exp/Reb may form a complex with other proteins (Discussion and model), this result could be interpreted as if the mutant proteins also interact with the proteins of the complex, preventing them to interact with functional Exp/Reb proteins. We have introduced the reviewer's suggestion in the text. We have now changed the Fig 1H to show a more representative example in response to one of the minor concerns of Reviewer #3.

**4.** For measurement, some detailed info is needed, for example, what is your area of interest?

We are now detailing in the manuscript, in material and methods and in the figure legends the regions used for measurements.

Minor comments:

**1.** Fig. 2 and Fig. 3. how do you define the region of apical and basal? An apical marker is needed here. N is the total number of embryos or the number of sections in the same embryo?

We have repeated the experiments of quantification of accumulation of different chimeric proteins in apical and basal domains using markers to clearly identify apical (using E-Cadherin or Armadillo) and basal domains (using α-Spectrin or SrcGFP, which allows us to visualise the whole cell, or Perlecan, which accumulates in the basement membrane). We are now providing more extensive explanations in the Materials and Methods section.

We have changed Fig 2E-G and Fig 3K-O to better represent the analysis and to show the quantification accordingly.

In figure legends we are indicating that the n corresponds to the number of ratios calculated (apical/basal level ratio per cell) used for quantifications and we are also indicating the number of embryos analysed in brackets.

**2.** Fig. 5H What is your area of interest to measure vesicles? Which tracheal segment do you measure? Some details need to be provided here.

In Fig 5H we evaluate number of vesicles in salivary glands. We analysed the whole salivary gland by obtaining a projection of 13 sections, centered in the luminal region. The details can be found in the Materials and Methods section, and for clarification we now show an example in Fig S2E

**Reviewer #2**

Major comments

1.) The authors nicely show that Kkv is able to synthesize chitin in a constitutive manner and that it can accumulate intracellularly. However, this needs some more input to understand the underlying biological sense. For example, what are the chitin vesicles' nature of early vesicles (st 13) and the unusual late (st15) chitin vesicles? Exocytosis, endocytosis or recycling? This can be clarified to understand chitin translocation and that synthesis and translocation are uncoupled. The authors tested rab5DN mutant salivary glands to exclude endocytosis. However, the chitin-positive vesicle size and amount in the rab5 mutant appear different from the control experiment, where much more intracellular chitin accumulates. Thus, it may suggest that some chitin vesicles are independent of Rab5-mediated endocytosis, others probably not. Indeed, the authors identified some KKv and some other chitin vesicles in all discussed intracellular processes; however, additionally, chitin appears to accumulate also in the cytoplasm. The authors conclude that Kkv protein might be able to polymerize chitin at all different intercellular stages, including endocytosis and degradation pathway. First, I wonder why chitin was found within membranous vesicles and, at the same time, within the cytoplasm. Second, does it make sense in the biological context when tracheal cells or other chitin-producing organs want to secrete chitin at the apical membrane while Kkv has the ability to produce chitin in all cellular areas, even in endosomes?
In this context, another fundamental question concerning chitin secretion and subsequent organization could be investigated with the author's tools. Are the chitin vesicles loaded with chitin binding proteins or deacetylases that organize the formation of the nano and makro fibrillar chitin matrix in tracheal tubes? For example, previous research showed a reduced luminal accumulation of the 2A12 antigen in kkv mutants and expRNAi knockdown embryos.

The reviewer raises different interesting points in this comment.

-Nature of chitin punctae: The reviewer wonders why chitin is found both in the cytoplasm and in vesicles that correspond to different intracellular trafficking stages.

We apologise that we did not make this point clearer in the original version and it has generated confusion. Prompted by the interesting comments of the reviewer, we have revisited this issue and we have looked again at our data in an attempt to describe in more detail chitin punctae with respect to GFP-Kkv. We have realised that most (if not all) GFP-Kkv+chitin common punctae actually show partial colocalisation, rather than perfect colocalisation. This observation has led us to propose a model to explain the nature of single chitin punctae and chitin+GFP-Kkv common punctae. We are now discussing this model in the Discussion section and we have corrected the text in the results section according to these observations.

Our hypothesis is that Kkv is able to polymerise chitin, but this chitin can only be translocated when Exp/Reb are present. Our colocalisation analysis indicates that Kkv is secreted, reaches the membrane, is internalised and either degraded or recycled back to the membrane. Throughout all this intracellular trafficking, Kkv must be localised in intracellular membranous vesicles with the catalytic domain (which polymerises chitin) facing the cytoplasm, according to the topological organisation of Kkv protein proposed in the literature. Thus, Kkv may be able to polymerise chitin intracellularly generating chitin fibers facing the cytoplasm. As indicated, we have realised that most (if not all) GFP-Kkv+chitin punctae show partial colocalisation. This result can be interpreted as if the chitin we detect in these Kkv vesicles corresponds to short polymers of chitin that are being synthesised facing the cytoplasm by Kkv in a constitutive manner. Because this chitin is not translocated (due to the absence of Exp/Reb activity), it is then released in the cytoplasm giving rise to the free chitin punctae we detect. This hypothesis perfectly fits with the live-imaging we are presenting (Fig 4O, S4G, and movies), where we can identify partial colocalisation of Kkv and chitin (corresponding to chitin synthesis) and the separation of punctae (corresponding to release of chitin).

-Nature of chitin punctae: The reviewer wonders whether it make sense in the biological context when tracheal cells or other chitin-producing organs want to secrete chitin at the apical membrane while Kkv has the ability to produce chitin in all cellular areas, even in endosomes?

We can only speculate about the biological meaning of chitin production. We are showing in this work that Kkv has the capacity to polymerise chitin intracellularly, strongly suggesting a constitutive activity of Kkv in chitin polymerisation. On the other hand, we also find that the endogenous levels of Kkv are not able to produce sufficient levels of chitin intracellularly that we can detect. Altogether this suggests that in normal conditions, while Kkv can polymerise chitin intracellularly, this may represent a residual activity.

-Nature of chitin punctae: Role of Rab5-mediated endocytosis

We blocked endocytosis using Rab5^DN^ expression to determine whether intracellular chitin derived directly from chitin synthesis in the cytoplasm or from chitin that was first translocated and then internalised. In addition, the experiment could also be used to test whether preventing Kkv internalisation and endocytic trafficking, precluded chitin polymerisation. Our results indicated the presence of intracellular chitin punctae when endocytosis is compromised. The chitin punctae that we identified were comparable to those observed when endocytosis is acting normally. In this respect we have already improved the images (using single sections or projections of only few sections) to show that in the absence or presence of endocytic activity, abundant chitin punctae are detected in the cytoplasm.

The results with Rab5^DN^ favour our previous hypothesis that chitin punctae may derive from short polymers synthesised by Kkv that have not been translocated and are instead released in the cytoplasm. In this case, most of these short polymers would be synthesised at the level of the apical cell membrane, where Kkv is strongly accumulated.

-Nature of chitin punctae: Presence of chitin binding proteins and deacetylases in the chitin punctae.

We have investigated the presence of deacetylases (i.e. Vermiform and Serpentine) and other chitin binding proteins (i.e. Gasp, visualised by 2A12 antibody) in intracellular chitin punctae in the trachea and salivary glands. For the experiments in the trachea, we used embryos at early stages (13/14) overexpressing Kkv to visualise chitin punctae in tracheal cells. We did not observe colocalisation of these chitin punctae with deacetylases or with Gasp. For the experiments in salivary glands, we used embryos expressing Kkv and Vermiform and Serpentine (which are not expressed in wild type salivary glands). We did not observe consistent colocalisation of chitin punctae with the deacetylases. These results indicate that chitin punctae in the cytoplasm may correspond to "naked" chitin fibers. We speculate that while deacetylases and Gasp should be able to bind intracellular chitin thanks to their chitin binding domains, they cannot do it because they accumulate in different cellular compartments. Chitin largely accumulates freely in the cytoplasm while the chitin binding proteins may be normally secreted to the extracellular space following the secretion pathway. These results are now described in the results section and shown in Fig S4B-F

**2.)** Observation of Extracellular kkv-GFP: does extracellular anti-GFP staining co-localize with the anti-Kkv antibody?

We performed this experiment. The results show colocalisation between GFP and Kkv antibody. This indicates that extracellular GFP detected corresponds to Kkv-GFP protein. This result is now shown in Fig S4H and described in the text.

**3.)** Putative Kkv microvesicles: the authors state that extracellular GFP staining could be Kkv located in microvesicles. I wonder whether the observed extracellular GFP puncta contain a membrane or other membranous proteins.

We agree with the reviewer that this is an important point. We have now repeated the experiments using CD4mIFP, a membrane marker. The results indicate that extracellular GFP-Kkv punctae correspond to membrane structures. This result is now shown in Fig 5E and described in the text

**3.)** Fig.1:
- general remarks: some images of this figure could be improved by showing the single channels of CBP to judge whether chitin is secreted and/or vesicles appear.

We are now showing only the single CBP channel in Fig 1F, 1N

-In addition, some images show higher magnifications, others overview only. It would be beneficial to visualize the small vesicles additionally with higher magnifications.

We are now showing insets for clarification in 1B',1F,1H

- Fig.1M: This image is problematic due to the epidermal background staining. The tracheal system is hard to recognize. A single channel of Cbp ist not indicated.

We have tried to improve the image to better show the tracheal system. We are also showing an inset of the trachea where the absence of luminal and intracellular chitin can be appreciated. The single CBP channel is not very informative in this case because no chitin is produced.

- Fig. 1P: Apical membrane marker or any cytoplasmic marker would be extremely useful to judge subcellular Exp localization in this experiment - this image is hard to compare with Exp localization in Fig S2D.

We are providing a better image to show the localisation of MH2-exp in the trachea and salivary glands, Figs 1P,Q, using an apical marker (Kkv in this case)

Fig. 2O: Apical/Basal accumulation, what are the numbers at the Y-axis?

The numbers correspond to the number of ratios calculated (apical/basal level ratio per cell) used for quantifications and in brackets we indicate the number of embryos analysed. We are now providing a better description in the Materials and Methods and in the Figure legend of the experiments. We are providing new quantifications of the apical/basal accumulation according to the suggestions of the reviewers.

**Fig. 3F:** The authors state that the WGTRE domain is required for ER exit of Kkv based on colocalization studies with KDEL and FK2. However, the study with FK2 is not convincing as immunostainings are of poor quality. The GFP construct appears not to be expressed in all tracheal cells, and moreover, the FK2 staining is faint. Thus, judging whether the protein is not ubiquitinated from the presented image is challenging. However, it does not change the key message, Kkv does not exit ER. By the way, there is a new paper showing Serca to be essential for ER exit of Kkv, which would fit the discussion of the kkv domains.

We agree with the reviewer that the image we initially provided was not very convincing. We have repeated the experiment, and we could confirm the previous result. We did not detect accumulation of FK2 in tracheal cells. Unfortunately, the FK2 antibody stainings are not excellent. However, we consider that the result is informative.

To determine FK2 staining in tracheal cells we used single sections instead of stack projections (to avoid background or unspecific labelling). For this reason, the GFP is not detected in some regions, because the tubes are normally wavy and cannot be visualised in a single section.

We are presenting a new image in Fig 3F where we think that the lack of FK2 can be better appreciated.

As the reviewer pointed, recent work identified a sarco/endoplasmic reticulum Ca^2+^-ATPase, Serca, that physically interacts with Kkv and regulates chitin deposition (Zhu et al., 2022). This information was already provided in the first chapter of the Discussion section. As the reviewer points, Serca may play a role in the WGTRE-dependent ER exit of Kkv that we detect.

**Fig. 7 -** model: First, Since the authors do not show that Kkv is part of a membranous microvesicle, I'm skeptical whether this should be part of a model that explains the shown data. Therefore, I'm asking the authors to delete it or to show it more clearly. Second, the meaning of the yellow arrowheads is not indicated. Third, the explanation in the legend is sound, but showing the two options could be improved.

Our results indicate that the Kkv microvesicles that we detect are membranous structures. Therefore, we would maintain this representation in the model.

The yellow arrowheads indicate the closed conformation of Kkv that prevents chitin translocation. It is represented as a plug. This has now been added in the model.

We have also added a third possible model as proposed by Reviewer #3

We have re-written the figure legend and we believe that the text is now more informative.

Minor comments:
**1.)** Missing reference: Chirin has been recognized importance in physiology (Zhao et al., 2019; Zhu et al., 2016) but also as a biomaterial (? Reference).
Suggestion: DOI: 10.3390/ma15031041
(Improving Polysaccharide-Based Chitin/Chitosan-Aerogel Materials by Learning from Genetics and Molecular Biology) This paper discusses the current usage and potential of chitin as a biomaterial in many disciplines.)

We thank the reviewer for the suggestion. We have introduced this reference in the text.

**2.)** 3.1, second paragraph final sentence: double point

This point has been corrected

**Reviewer #3**

Major concerns

- A major conclusion of the paper is that Exp/Reb are not required for chitin synthesis. On the most basic level this statement is well supported, because chitin grains are made in the cytoplasm in the Exp/Reb mutants. However, I think the field would be better served with a more nuanced consideration or the role of Reb/Exp. From the data presented, it seems that in the absence of Reb/Exp, the total amount of chitin produced is greatly reduced. I think it would be worth considering Exp/Reb, or the synthesis process in general, as having processivity or duty cycle or quality control such that in the absence of Exp/Reb while Kkv may make short chitin polymers, or occasional long polymers, the major production of chitin doesn't get going without Exp/Reb. Thinking of Reb/Exp as processivity factors in addition to export factors dramatically changes how one thinks of the proteins and the process of chitin synthesis. While these considerations can be handed with some discussion, it would be very interesting to look at the length of the chitin polymers in the Reb/Exp mutants and see if the average chain length is much reduced. This would help distinguish between Exp/Reb reving up the total number of Kkv molecules that produce chitin and Exp/Reb allowing the same number of Kkv molecules to stay active and produce much longer chitin chains. A caveat here is that I have no idea how hard this is to do, so I won't put this at the level of a required revision, but this result would significantly deepen the analysis in the paper.

The comments of the reviewer are extremely interesting and we thank her/him for this insightful point of view. However, as the reviewer acknowledges, these hypotheses are very difficult to address and for this reason she/he is not asking for these experiments in a revised version. Investigating the issues that she/he raises would require a full and detailed analysis that is beyond the scope of this manuscript, but may represent the entry point for our future investigations.

In this work we identify and investigate a role for Exp/Reb in promoting chitin translocation, but we cannot exclude additional activities of these genes (probably in coordination with a protein complex). We have now introduced some of the reviewer's interesting observations and contributions in the discussion.

Importantly, a very recently published paper that analyses the structure of the *Phytophthora sojae* chitin synthase, Chs1, has identified a "gate lock" in its chitin-translocating channel. This "gate lock" ensures the growing of the oligomer and directs the polymer through the exit of the channel towards the extracellular domain. The domain serving as the "gate lock" is highly conserved among CHS (Chen, W., Cao, P., Liu, Y. *et al.* Structural basis for directional chitin biosynthesis. *Nature* (2022). https://doi.org/10.1038/s41586-022-05244-5). It is tempting to speculate that Kkv may act in a similar manner, so that these recently published results provide support to our model. In this scenario, Exp/Reb would play a key central role regulating this "gate lock", and therefore could act both as processivity factors and translocating factors. We have added this information in the Discussion section.

- In looking that the subcellular localization of the Kkv and Reb in regular and super resolution, the authors I think the authors missed an important, but straight forward way to gain insight into the apparent complementary distribution of Kkv and Exp/Reb. In stage 16 WT embryos, Kkv has a distinct ringed pattern that corresponds to the tanedial ridges (e.g. clearly visible in Fig. 6A and 6G). How those ridges are set up is unclear, although there are some interesting Turing-pattern models out there. One prediction might be that Exp/Reb should be in between the Kkv rings. If so, maybe Exp/Reb are key components of patterning chitin secretion to make this 3D patterned matrix? Alternatively, maybe Exp/Reb act on a smaller length scale and will match the Kkv ring pattern, just not overlapping with Kkv at the very fine scale. These are straightforward experiments and again could provide key insights into the function of Exp/Reb.

We have analysed the pattern of Kkv and Reb at late stage 16 embryos, when Kkv clearly accumulates in stripes that correspond to the taenidial folds. Our results indicate that Exp/Reb seem not to be involved in establishing this supracellular patterning. We find that Reb shows a complementary pattern to that of Kkv at a fine scale (e.g between discontinuities in Kkv pattern along the stripe), rather than accumulating in complementary stripes to those of Kkv. This suggests that Exp/Reb play a role in modulating the pattern of Kkv distribution at the local subcellular level. This result has been added to the text and it is shown in Fig 7B.

- In general, most of the figures do not include WT or a control for comparison. This makes it hard for non-experts to assess what the effect of a mutation or condition is. For example, there are no examples of WT or Df(exp reb) in Figures 1-4. I realize this would increase the number of panels, but the paper would be more accessible if comparisons were within figures instead of comparing between main and supplementary figures and other papers.

We understand the reviewer's point and we agree with her/his comment. However, introducing the control in Figs 1-4 would enlarge the figures significantly, which we believe to be already very big. In case the editorial agrees in the arrangement of most images from supplementary figures to the main figures, we would be willing to address this request from the reviewer.

- To bolster the case the Exp/Reb directly regulate Kkv distribution, the authors should examine the distribution of Kkv in a catalytically null Kkv mutant, or drugs that block Kkv, or mutations in other genes required for Kkv activity to show that the altered distribution of Kkv in Exp/Reb mutants is a direct consequence of the lack of Exp/Reb rather than in indirect consequence of lack of extracellular chitin, which causes gross perturbations in the trachea. Also, are there differences in the distributions of Kkv in salivary glands with or without the presence of Exp/Reb? If Exp/Reb change the distribution of Kkv in the salivary glands, which normally do not express Kkv and presumably many other components of the chitin ECM system, this would be a powerful argument that there is a direct effect.

We agree with the reviewer that this is a very important point. As the reviewer indicates the lack of chitin and associated defects in exp reb mutants could be the primary cause of Kkv apical distribution defects. To discard that the lack of chitin on its own (independent of Exp/Reb activity) affects Kkv's apical distribution we attempted different approaches. We aimed to analyse Kkv apical distribution in kkv catalitically null mutants. However, we found that Kkv does not localise apically in these mutants (Fig 5B), in agreement with the fact that Kkv activity and localisation are coupled. We then resorted to use stage 15 embryos to analyse Kkv pattern. At this stage chitin is still not accumulated to form a cuticle, which we reasoned may directly impact the apical membrane, and instead is found in a filament that fills the lumen of the tubes (although we cannot discard that the luminal chitin filament affects the apical membrane of tracheal cells).

The experiment that the reviewer proposes is an ideal one. Salivary glands do not normally deposit chitin, but they can do it when they are forced to (e.g. expressing Kkv and Exp/Reb). We found that salivary glands express kkv at certain levels, but not exp or reb. So, we compared the pattern of apical Kkv distribution in control salivary glands and in salivary glands where we ectopically expressed reb. As described now in the text, the analysis of the spatial Kkv distribution under these conditions indicated that the presence of reb modifes the Kkv pattern, supporting the notion that Exp/Reb regulate Kkv apical distribution. Interestingly, the analysis of control salivary glands where exp reb are absent, shows a tendency of Kkv distribution towards spatial randomness, similar to the observation in the trachea of exp reb mutants.

The pattern of Kkv distribution upon expression of reb in salivary glands does not restore the order of Kkv apical distribution observed in wild type trachea, although it modifies the pattern of Kkv. We suggest that the high levels of reb may generate an artefactual situation that does not allow mimicking of the non-random pattern that Kkv presents in the trachea where all proteins are produced at their wild type levels. In addition, salivary glands (normally a non-chitin depositing tissue) may lack other elements or may have specific apical membrane particularities that could also affect Kkv’s apical distribution. Nevertheless, the important conclusion from our experiments is that, in different tissues analysed, the presence of Exp/Reb regulate the apical distribution of Kkv. These experiments, results and conclusions are now added in the text and presented in Fig S5A-H

Minor concerns.

- Page 5 "These intracellular chitin punctae disappeared from stage 14, when chitin is then deposited extracellularly (Fig 1B')." Fig. 1B' is stage 15 embryos.

The reviewer is right in this observation and we have corrected the text

- Page 5 "lead to tracheal morphogenetic defects". It would be helpful to the reader if the text or legend told the reader what they were looking for? Broken tubes? Inflated tubes? Variable tubes?

We have added a description of the defects in the legend of Fig S1

- Fig. 1H. Main text says "co-expression of Kkv and expΔMH2/rebΔMH2 did not lead to tracheal morphogenetic defects (Fig 1H, ...". The tracheal dorsal trunk in Fig. 1H does not look WT. The legend does not state the stage, but the DT looks to have an enlarged diameter and it might be too long. Please present measurements on stage 16 trachea to confirm that there is no effect on tracheal morphology.

As requested by the reviewer we have quantified tube size of mutant (btlGal4>UASKkvGFP; UASExp^ΔMH2^) and control embryos (yw embryos processed together with the mutants) at stage 16, stained with CBP to visualise the tracheal tubes.

We have quantified tube length in the dorsal trunk (main anterio-posterior branch) using confocal projections. We traced the path of the dorsal trunk using the freehand line selection tool of Fiji software between the junction dorsal trunk/transverse connective from metamere 2 to 9. The length of embryos were measured using the straight line selection tool of Fiji software. Dorsal trunk length was calculated as the ratio between the dorsal trunk path length and the embryo length.

Confocal projections were also used to analyse the diameter of the embryonic dorsal trunk. In this case, we traced a line anterior to the dorsal branch in metamere 8.

As it can be appreciated from the quantifications, the length and diameter of mutants are not significantly different from the control, indicating no major defects in tracheal morphology. We note, however, a slight, non-significant, increase in the length and diameter in mutant conditions. This could be due to a mild effect on chitin deposition, in line with a possible effect of UASExp^ΔMH2^ interfering with endogenous Exp, as suggested by Reviewer #1 (major point 3). Because we do not find a significant effect in tube size and the analysis of tube size is not a major point in this work, we are not adding this information in the manuscript.

We have changed the image in Fig1H to show a more representative example.

- Fig. 3E there is a lot of GFP-Kkv that is not in co-localized with the KDEL marker. Can the authors clarify what compartment all the other staining is? ER?

In the image shown in Fig 3E only the green cells correspond to the tracheal cells and are the ones we are using to investigate colocalisation with the ER marker KDEL (in red). KDEL is also labelling the ER of other cells. We have improved the image

- Section 3.1. The authors imply that the WGTRE domain is specifically required for ER exit. However, an alternative is that absent the WGTRE domain, the protein just does not fold correctly, which would also preclude ER exit, but would be a different problem for the protein to make chitin if it isn't folded.

We used the FK2 antibody that recognises ubiquitination to address the point that the reviewer has made. Because we do not find colocalisation between GFP-Kkv^ΔWGTRE^ and FK2, our results suggest that GFP-Kkv^ΔWGTRE^ can still fold but not exit the ER. This information is found in the text and Fig 3F.

- Page 15. I disagree with statement "At stage 16, control embryos showed a highly homogeneous apical distribution of Kkv in stripes, corresponding to the taenidial folds, and Kkv vesicles were largely absent (Fig 6G)." In Fig. 6G, the tandeal ring pattern is clearly visible, as are the fusion cells. If Kkv distribution were "highly homogeneous" these structures/pattern would not be visible.

The reviewer is right in this observation and we have corrected the text

- Page 15. I also disagree with the characterization of the apical Kkv distribution in st 15 embryos. "In control embryos we detected a very uniform and homogenous pattern of apical Kkv (Fig 6I).". To my eye, the pattern is punctate and random for the clumps of stain, with the underlying beginnings of the tanidial pattern starting to be visible. The pattern appears neither uniform nor homogenous.

We agree with the reviewer that we misused the terms “uniform” and “homogeneous” to characterize the pattern of Kkv’s apical distribution at that stage. We have corrected the text and defined more accurately the patterns we observed.

- P16. The degree of order in the distribution of Kkv is overstated. The authors state that "The results of this analysis, showed that Kkv on the apical membrane, is evenly distributed following a regular pattern (Fig. 6L,L',L',M)." However, given that there is barely a visibly perceptible difference between the actual distribution of Kkv in 6L' and a calculated random distribution in 6L", and that the pattern is neither visibly even or regular, it would be more representative to say something to the effect that the analysis shows there is "underlying order" or "some degree of order" or a "non-random pattern". Visually, the key difference between 6L ' and L" is that there are fewer closely clustered Kkv dots. You could still have an uneven distribution of Kkv that maintains minimum spacing, which is a kind of ordered organization, but not one that would be assumed from the description. It would be helpful if the authors instead of just saying a "regular pattern" also stated the nature of the pattern they observe, i.e. Grid? Stripes? Minimum spacing?

We have modified the text according to the reviewer’s suggestions.

- Discussion. Another model for the role of Exp/Reb could be to bind and neutralize an inhibitor of Kkv activity. This would account complementary distribution of Kkv and Exp/Reb.

We agree with the reviewer that this is also a possible model. We have added this model in the Discussion section and in the model in Fig 7.

- Fig. 6L. what tissue is being analyzed? Presumably trachea, but this should be specified as salivary glands are also mentioned in the legend.

We have added in the legend that the analysis corresponds to tracheal cells

- Fig. 7 C models. I believe that the super resolution data is not accurately accounted for in the models. In both model 1 and model 2, Kkv and Exp/Reb are shown to be in close proximity, but the super resolution data suggests that most Kkv and Exp/Reb are separated hundreds of nanometers. Further, showing Kkv and Exp/Reb as touching was not supported by the coIP experiments, which failed to detect an interaction. It is possible that only a small fraction of Exp/Reb that is in close proximity to Kkv is active, but if so, this should be explicitly mentioned in the models to reconcile the data showing that Kkv and Exp/Reb are mostly not anywhere near each other.

The reviewer is right in this observation and we have modified the model accordingly to show that Exp/Reb and Kkv are not in contact

- -Image analysis. Please detail the criteria for "apical" and "basal" regions were the basis for freehand segmentation. What was counted as apical and what was basal?

This point has also been raised by other reviewers. In this revised version we have changed the figures according to the new quantification using apical, basal and cell markers. We have also included additional information in the Figure Legends and Materials and Methods sections to better describe the analysis.

- Abstract and Introduction: The authors state that "We find that Kkv activity in chitin translocation, but not in polymerization, requires the activity of Exp/Reb, and in particular of its conserved Na-MH2 domain.", but then follow that with the statement that "Furthermore, we find that Kkv and Exp/Reb display a largely complementary pattern at the apical domain, and that Exp/Reb activity regulates the topological distribution of Kkv at the apical membrane." Many readers, will find the use of "furthermore" confusing because they will take furthermore as the about to be described data logically following the previous data, but then run headlong into the fact the Kkv and Exp/Reb show a complementary distribution, which does not obviously follow from Kkv activity requiring Exp/Reb. The authors could clarify this and highlight the interesting, unexpected and exciting nature of their results by replacing "Furthermore" with "Unexpectedly" or "Surprisingly", and emphasizing the important role of Exp/Reb in Kkv organization. Maybe something like: Unexpectedly, we find that although Kkv and Exp/Reb display largely complementary patterns at the apical domain, Exp/Reb activity nonetheless regulates the topological distribution of Kkv at the apical membrane.

We thank the reviewer for this observation and we have changed the text accordingly in the abstract and last paragraph of the introduction
