# Supplementary material for: A dynamic interplay between chitin synthase and the proteins Expansion/Rebuf reveals that chitin polymerisation and translocation are uncoupled in Drosophila
Source: PLoS Biol. 2023 Jan 23;21(1):e3001978. doi: 10.1371/journal.pbio.3001978 (PMC9894549; doi:10.1371/journal.pbio.3001978)
Supplement: Attachment — Submitted filename: Response to reviewers.docx [file pbio.3001978.s012.docx]

**Response to reviewers:**

Reviewer 1:

There is a minor comment regarding the expression pattern of endogenous KKV and GFP-KKV.
Figure S1. (C) In wild type embryos, endogenous KKV is apically localized, almost no intracellular KKV is observed. (d) in btl> GFP-KKV, in addition to apical localization, vesicular KKV-GFP is clearly observed.

These intracellular KKV-GFP vesicles might not contribute significantly to intracellular chitin in exp reb mutants, however, this observation should be mentioned.

The reviewer is right with this observation. We are now including this observation in the text of the legend for the S1 Figure. In addition, we have added arrows in the Figure to better document this observation.
